# Analysis of the Spatial Relationship between Ecosystem Regulation Services and Rural Tourism

**DOI:** 10.3390/ijerph20053888

**Published:** 2023-02-22

**Authors:** Junying Wang, Guifeng Han, Jing You, Liang Zhu, Yafei Li, Xiang Zhou

**Affiliations:** 1 Observation and Research Station of Ecological Restoration for Chongqing Typical Mining Areas, Ministry of Natural Resources, Chongqing Institute of Geology and Mineral Resources, Chongqing 401120, China; 2 College of Architecture and Urban Planning, Chongqing University, Chongqing 400044, China; 3 Chongqing Tongnan District Land Reserve Renovation Center, Chongqing 402660, China

**Keywords:** rural vitalization, value realization of ecological products, ecosystem regulation services, rural tourism, driving effects

## Abstract

In the context of China’s implementation of the rural revitalization strategy and establishment of a mechanism to realize the value of ecological products, rural tourism as an eco-friendly industry promotes regional social and economic development with high-quality natural and ecological conditions in rural areas and is one of the key patterns to realize regional green development. Existing studies in the field focus on the spatial relationship between rural tourism and traditional factors such as economy, population, and transportation and ignore to some extent the relationship between ecosystem services and rural tourism. However, from the perspective of distribution, rural tourism is popular mainly in areas with high ecological quality, so it can be inferred that there is a relationship between ecosystem services and rural tourism. Therefore, this paper targets the key problem—the spatial relationship between ecosystem regulation services and rural tourism, taking rural tourist spots in six districts and counties in the Wuling Mountains in southeastern Chongqing as the subjects, and using the geo-econometric analysis method and geographic detector model to analyze the spatial-driving and development-support roles of ecosystem services on rural tourism. The results show that: (1) the nearest neighbor index of the distribution of rural tourist spots in the regions of study is 0.28, showing a significant clustering trend as a whole; (2) there are obviously high-value areas among the six types of ecosystem regulation services, which are mainly distributed in forest ecosystems; (3) the driving effects of dual factors are more significant, and the combined driving effects of climate regulation and anion supply services are the most significant, with the q value of these driving effects being 0.1962; (4) from the perspective of the supply–demand relationship of industrial development, ecosystem services play an important role in supporting the development of rural tourism. Based on these results, it is proposed in this paper that, in the next step, a comprehensive analysis of the impact of ecosystem regulation services should be conducted during the special planning of rural tourism and the rational layout of industries should be guided on the premise of compliance with space-use control, as well as economical and intensive land use, to support the scientific formulation of regional rural tourism development strategies with new ideas based on basic analysis and better realize the value of ecological products and boost rural revitalization.

## 1. Introduction

As a typical business development pattern of industry integration in rural areas, rural tourism is an eco-friendly industry that aims at realizing sustainable development with the high-quality natural and ecological conditions of rural areas. The development theories, spatial distribution laws, and community effects of rural tourism are continuously attracting academic attention. Chinese scholars have proved through analyses of existing practices that rural tourism can increase the income of farmers and make them rich [1,2], optimize the industrial structure of rural areas [3], and boost rural revitalization [4,5]. Over the past few years, China put forward the strategy of rural revitalization and the policy of establishing the mechanism for realizing the value of ecological products, and all local governments actively responded to the call and vigorously expanded their rural tourism industries to realize the value of ecological products and boost rural revitalization. In Chongqing, for example, as stated in the 2020 Tourism Statistics Bulletin of Chongqing City, 29 villages were identified as key destinations of nation-level rural tourism, 50 villages were identified as key destinations at the municipal level, 200 more traveling routes of rural tourism were established, and the city recorded consolidated income from tourism of RMB 65.8 billion, with its economic development effectively advanced while the environment was well-protected.

From the 1990s until now, scholars worldwide have carried out a great many studies on rural tourism [6,7]. In recent years, as rural tourism scaled up greatly, studies in this field began to focus on the spatial distribution and layout of rural tourism, as well as the relevant influencing factors [8,9,10,11,12,13,14], development patterns and theories [15,16,17], and community effects [18,19,20]. Among them, the spatial distribution and layout of rural tourism and the relevant influencing factors have become the focus of the academic circle in recent years. Most scholars used methods such as the nearest neighbor index and kernel density analysis [9,10,11,12] to conduct systematic analysis of the spatial distribution characteristics and the relevant driving factors of the key villages and tourist spots of China’s rural tourism, and they found that the traditional factors, including the distribution of A-level scenic spots, population density, and road network density, significantly affect the spatial distribution of rural tourism. However, there is a lack of attention to the relationship between good ecological environments and the spatial distribution of rural tourism.

Since the end of the 20th century, studies on the evaluation and accounting of ecosystem services have attracted more and more attention from scholars. Scholars, such as Costanza [21,22], Ouyang Zhiyun [23,24], Fu Bojie [25,26], and Xie Gaodi [27,28], have successively carried out in-depth studies in the aspects of amount estimation, value assessment, and practical applications of ecosystem services. After years of development, a sophisticated and complete system for the estimation and accounting of ecosystem services has been formulated, and it has been widely applied in the practice of the estimation and accounting of ecosystem services [29,30,31,32,33], appraisal of ecological conservation [34,35], and the analysis of different ecological patterns and partition of ecological functions [36,37,38,39]. Notwithstanding that the existing definition and connotation of ecological products is still controversial, it is generally believed that “ecological products” in a generalized sense shall mean ecosystem services with externalities [40]. For this reason, the monetization of the value of ecosystem services is also a key way to realize the value of ecological products.

The results of the preceding studies show that scholars mainly focused on traditional indicators, such as economy, population, and traffic [9,10,12,13], in analyzing the influencing factors for the spatial distribution of rural tourism. There is still a lack of research on the relationship between the spatial layout of rural tourism and the distribution of ecosystem services. To some extent, we have neglected the influence of high-quality services brought to humans by the ecosystem (namely, ecological products) on the spatial distribution of rural tourism. However, from the perspective of distribution, rural tourism is popular mainly in areas with high ecological quality, so it can be inferred that there is a relationship between ecosystem services and rural tourism. Therefore, targeting the spatial relationship between ecosystem regulation services and rural tourism, the authors of this paper, from the perspectives of rural revitalization and value realization of ecological products, used POI (point of interest) capture technology [41,42,43], a data collection means that has been widely used by scholars in recent years, to obtain data concerning the distribution of rural tourist spots in the six districts and counties of the Wuling Mountains in southeastern Chongqing, and also used the geographic detector model to probe into the driving effects of ecosystem regulation services on the spatial distribution of rural tourism and further reveal the functional mechanism by which ecosystem services facilitate rural tourism development, in the hope of providing a basis for scientific decision-making in formulating the strategies for next-step regional rural tourism development and offering new ideas of basic analysis for the spatial planning of relevant tourism development to better realize the value of ecological products and facilitate the implementation of the rural revitalization strategy.

### Overview of the Researched Areas

**Figure 1:**
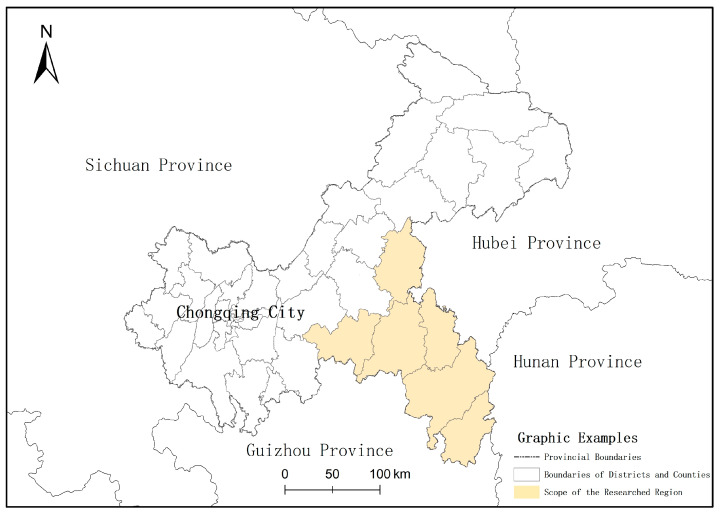
Scope of the Researched Region.

The Wuling Mountains are located in the southeastern part of Chongqing City. In this study, the authors select six districts and counties (autonomous counties), which are the most representative of Wuling Mountains (Figure 1), as the regions to be studied, namely Qianjiang District, Wulong District, Shizhu County, Xiushan County, Qiuyang County, and Pengshui County. They cover a total area of 19,800 km^2^ and are within the scope of east longitude 107°14′–110°11′ and north latitude 29°39′–32°13′. The whole region is characterized by abundant natural and ecological resources and good environmental status. The Overall Planning of the Space of National Land of Chongqing City (2021–2035) clearly indicates that in the future the integrative development of culture and tourism will be the focus of work. This region will be built into a new benchmark for the integrative development of culture and tourism and serve as an important tourism development region of Chongqing City.

## 2. Data Source and Research Method

### 2.1. Data Source

In this study, the authors set the existing rural tourist spots of six districts and counties in the Wuling Mountains of southeastern Chongqing in 2019 as the research targets. Through keywords such as “rural tourism”, “blossom valley”, “countryside villa”, “agritainment”, and “agricultural garden”, the authors obtained the POI point data that comply with the definition of rural tourism within the researched region in 2019 from the Amap platform. After “cleansing” these data through ways such as the deletion of duplicate values and information that does not comply with the definition of rural tourism, the authors obtained the POI point data of 1635 rural tourist spots within the researched region. The land utilization data that are used in this study come from the authorities of natural resources management; DEM (digital elevation model) data come from a cloud database of geographic and spatial data; data of rainfall, temperature, soil, etc. come from the national platform of shared service of scientific and technological resources; other data like negative ion monitoring and straw-mulching rate come from the statistical information of authorities such as the Forestry Bureau and rural villagers’ committees.

### 2.2. Research Method

#### 2.2.1. Analysis of Spatial Layout

The authors mainly adopt two methods of geographic measurement and analysis, the nearest neighbor index and kernel density analysis, to probe into the basic spatial layout of rural tourist spots within the researched region.

(1) The nearest neighbor index

In this study, the authors use the nearest neighbor index (*NNI*) to reflect the distribution types of factors within the researched region. The evaluation was undertaken mainly through the ratio between the actual average distance of the nearest neighbor factors and the idealistic average value of the shortest distance of factors that are randomly distributed.

(1) $$NNI=\frac{{đ}_{0}}{{đ}_{E}}$$

where ${đ}_{0}$ is the actual average value of the shortest distance of the nearest neighbor factors; ${đ}_{E}$ is the idealistic average value of the shortest distance of factors that are randomly distributed.

(2) Kernel density analysis

Kernel density analysis means calculating the probability of occurrence of the factors within the researched region in accordance with the bandwidth determined, so that the aggregation conditions of the factors within the researched region could be reflected more directly.

(2) $$F\left(x\right)=\frac{1}{nh}\overset{n}{\underset{i=1}{\sum }}k\left(\frac{x-x_{i}}{h}\right)$$

where *F*(*x*) is the probability of the factor appearing in a certain place; *h* is the search radius, namely, bandwidth; *n* is the number of factors; *k*(*) is the kernel function; (*x* − *x_i_*) stands for the distance between a certain factor and the *i*-th kernel.

#### 2.2.2. The Accounting of Ecosystem Regulation Services

The accounting method of ecosystem regulation services used in this study is mainly based on the accounting system of ecosystem services constructed by scholars such as Ouyang Zhiyun [30,44], as well as normative standards like the *Specifications for Assessment of Forest Ecosystem Services* (LY/T1721-2008). According to the relevance between the aforesaid accounting method and the distribution of rural tourist spots, as well as data accessibility, the authors choose six indicators, water conservation, soil preservation, carbon sequestration of the ecological system, oxygen provision, climate adjustment, and negative ion provision (see Table 1), to calculate the physical quantity of the various indicators of the ecosystem regulation services, and at the same time probe into the degree of its relevance with the spatial layout of rural tourism. In addition, based on the different precisions of the basic data, the authors used the minimum analysis unit—5 km × 5 km—to calculate water conservation and used the minimum analysis unit—1 km × 1 km—to calculate soil conservation, carbon sequestration of the ecological system, oxygen provision, climate adjustment, and negative ion provision.

#### 2.2.3. Geographic Detector

In this study, the authors mainly choose two models of the geographic detector for the analysis and research of the driving effects of ecosystem regulation services within the researched region on the spatial layout of rural tourism.

A differentiation and factor detector is used for the analysis of the interpretation power of a certain kind of driving factor on the spatial distribution characteristics of the factors. It is generally expressed by the *q* value [45].

(9) $$q=1-\frac{\sum_{h=1}^{L}N_{h}\sigma_{h}^{2}}{N\sigma^{2}}$$

where *q* means that a certain driving factor could explain the spatial distribution of *q* × 100% rural tourist spots within the researched region; *h* is the autochthonous variable of unit grid or the stratification of the driving factors; *Nh* is the unit number of the *h*-th layer; *N* is the all-region unit number; $\sigma \frac{2}{h}$ is the variance of the *h*-th layer; $\sigma^{2}$ is the variance of the all-region dependent variables.

On the basis of the *q* values respectively calculated through the two driving factors (*X_i_* and *X_j_*), the interaction detector recalculates the *q* values when the interaction occurs and then compares the interaction-related *q* values (*q*(*X_i_* ∩ *X_j_*)) with the *q* values driven by a singular factor, so that the interaction type of the two driving factors could be determined [46] (Table 2).

This study emphasizes the research on the relationship between the ecosystem regulation services and distribution of rural tourist spots. For this reason, six types of ecosystem regulation services were chosen as the driving factors. In addition, Jenks natural breaks was chosen for the discretization treatment of the data of driving factors (Table 3).

## 3. Result Analysis

### 3.1. Spatial Layout of Rural Tourism

In 2019, the NNI value of the rural tourist spots within the six districts and counties in the Wuling Mountains of southeastern Chongqing was 0.28 (Table 4), while the *p* value was 0, indicating that the credibility of the confidence interval was 100%. By referring to the classification standard given by Pan Jinghu et al. [11]. (NNI ≤ 0.5 stands for aggregated distribution. “0.5 < NNI ≤ 0.8” stands for aggregation-random distribution), the authors made the judgment that the rural tourist spots within the researched region demonstrated obvious features of aggregated distribution in terms of spatial layout.

**Figure 2:**
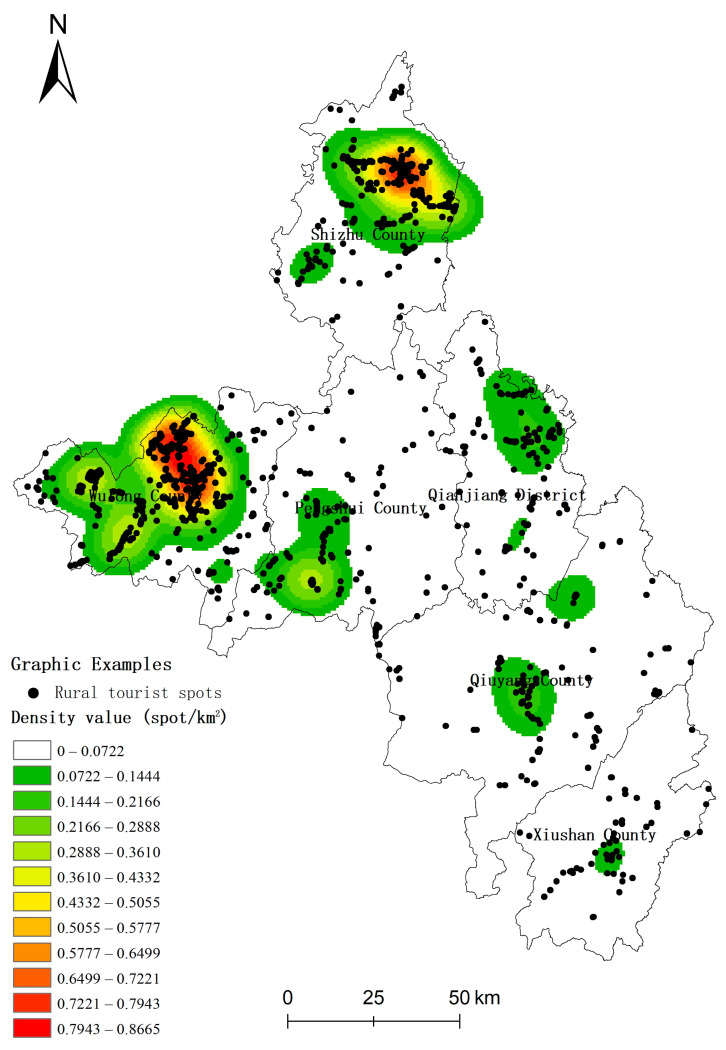
Kernel density estimation of rural tourism.

If the result of kernel density analysis (Figure 2) is taken into consideration, it could be discovered that the distribution of rural tourist spots within the researched region demonstrated multi-kernel and closely grouped distribution features. The areas with high-density distribution were mainly located in the central part of Wulong County and the northern part of Shizhu County and involved scenic spots like the Xiannv Mountain National Forest Park of Wulong District and Huangshui National Forest Park of Shizhu County, which had very good natural and ecological conditions. In addition, distribution features of sub-high density were observed in the southeastern part of Pengshui County, the northern part of Qianjiang District, and the central parts of Qiuyang County and Xiushan County. The distribution of rural tourist spots in the remaining areas was relatively regular.

### 3.2. Spatial Layout of Ecosystem Regulation Services

**Figure 3:**
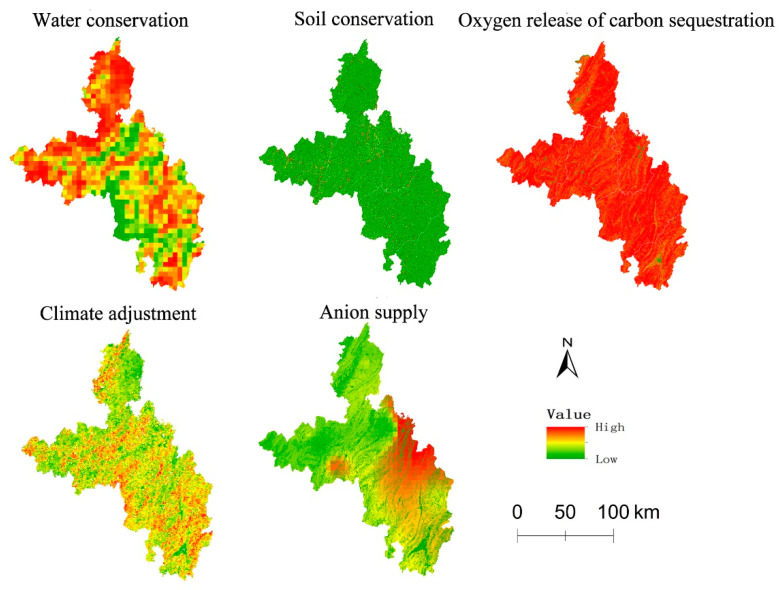
Distribution of ecosystem regulation services.

The results (Figure 3) reveal that in 2019 there was a drastic difference between the spatial distribution/layout and the ecosystem regulation services within the scope of the researched region. As far as the different types of ecosystem regulation services were concerned, there were many high-value areas in water conservation service, which were mainly located in the northern and western parts of the researched region that involved the watershed of the Yangtze River and its branches. There were a few high-value areas scattered in the southeastern part; no obvious rules could be observed in the overall distribution of the soil conservation service. The researched region mainly had the landforms of a mountainous area, and there was a high risk of soil erosion. For this reason, there were few high-value areas of soil erosion, and the distribution of them was decentralized; the oxygen supply and the ecological system were subject to similar spatial distribution rules. As the forest ecosystems that had remarkable functions of carbon sequestration and oxygen release within the researched region were scattered extensively, the distribution of high-value areas of the services of carbon sequestration and oxygen release was also extensive; the distribution of high-value areas of climate adjustment service was generally decentralized. For this reason, there were few high-value areas in the northern part of the researched region, and in the southern and central parts, the distribution of high-value areas was dense; the high-value areas of the anion supply service were mainly located in the eastern part of the researched region, and in the central part they were scattered very extensively. That was to say, there were many more high-value areas in the eastern part than in the central part.

Generally speaking, among the six types of ecosystem regulation services within the researched region, except for the soil conservation service, the other five types of services all demonstrated the distribution features of obviously high-value aggregation and displayed to some degree the features of aggregated distribution. Furthermore, a certain degree of relevance was observed between the spatial distribution of ecosystem services and the distribution of ecosystem. The rural tourist spots with high-value services were scattered in areas where the ecological functions such as the forest ecosystem were good.

### 3.3. Analysis of the Driving Effects of Space

#### 3.3.1. Differentiation and Factor Detection (Single-Factor Analysis)

Through the 5 km grids, the distribution of physical quantity of ecosystem regulation services within the researched region and the distribution of rural tourist spots were matched, and the results were obtained through the model analysis of the differentiation and factor detector (Table 5). The results show that, under the single-factor influence, the driving effects of the physical quantity of the six types of ecosystem regulation services within the researched region on the distribution of rural tourist spots were generally not strong. The average *q* values were lower than 0.1, while the *p* values of the three factors of soil conservation, carbon sequestration of the ecosystem, and oxygen supply were not 0. This means that the significance level of the result was low. The *p* values of the remaining factors were 0. This means that the confidence level of the result was high.

Relatively speaking, water conservation had the strongest driving effects among the six factors, while the *q* value was 0.0628, which means that the spatial distribution of 6.28% of the rural tourist spots could be explained. Climate adjustment and anion supply had the second strongest driving effects, while the *q* values were 0.0365 and 0.0333, respectively. Soil conservation had the weakest driving effects, while the *q* value was 0.0063, which means that the spatial distribution of only 0.63% of the rural tourist spots could be explained.

#### 3.3.2. Detection of Interaction (Analysis of Combined Factors)

On the basis of single-factor analysis, the interaction between factors was further analyzed, and the results are as shown in Table 5. As shown, under dual-factor effects, the interactions and mutual driving effects between the six driving factors in the regions of study were strengthened. Among the 15 interaction results (Table 6), the combination of carbon sequestration of the ecological system and oxygen supply had dual-factor-enhancing interaction, while others had nonlinear-enhancing interaction, indicating that the combined driving effect of these two ecosystem supply services in the regions of study was significantly stronger than the driving effect of a single factor on the spatial distribution of rural tourist spots, and that ecosystem regulation services have compound driving effects on the spatial distribution of rural tourism, and it is difficult for a single one of them to support the development of rural tourism.

Among them, the interaction between climate adjustment and anion supply features the strongest driving effect. The formula “*q*(*X*_6_ ∩ *X*_5_) = 0.1962” can be used to explain the spatial distribution of 19.62% of the rural tourist spots within the researched region. Four combinations, namely *q*(*X*_5_ ∩ *X*_3_) = 0.1815, *q*(*X*_5_ ∩ *X*_4_) = 0.1815, *q*(*X*_2_ ∩ *X*_1_) = 0.1712, and *q*(*X*_5_ ∩ *X*_1_) = 0.1623, have *q* values greater than 0.15, indicating that the driving effects of the interactions between such combinations as climate adjustment and carbon sequestration of the ecological system, climate adjustment and oxygen supply, soil conservation and water conservation, and climate adjustment and water conservation are also strong on the spatial distribution of rural tourist spots within the regions of study.

On the whole, among the combinations with significant interactions, the climate adjustment service enjoys the highest frequency, followed by the water conservation service, which corresponds to the results of single-factor analysis that the effect of water conservation is the most significant under the action of a single factor, followed by climate adjustment. This proves that, in the regions of study, these two services have significant driving effects on the spatial distribution of rural tourism and, conversely, that more rural tourist spots in the regions of study are located in areas with lower temperatures and richer water sources, which are closely related to the climate characteristic of long-lasting high temperature in summer of most regions in Chongqing, driving a large number of tourists to spend their summer holidays in regions with comfortable temperatures and abundant water sources.

### 3.4. Analysis of the Development and Support Effects

Through the analysis of the information regarding the 1636 rural tourist spots within the researched region, it could be discovered that 1480 of them were named “Agritainment”, “Countryside Villa”, “Mountain Villa”, etc., and the mainstream business pattern was “specialty catering services + accommodation”, which accounted for 90.52% of the rural tourist spots. The remaining rural tourist spots were mainly named “Fruit Garden”, “Agricultural Garden”, “Countryside Composite”, etc. Their business patterns were more diversified, which involved the picking and selling of specialty agricultural products, sightseeing, farming experience, camping, etc. All the aforesaid business patterns called for favorable natural and ecological environments. Specialty catering services and the picking of farm products required that there were vegetables, fruits, mushrooms, grains, etc. with local features, while accommodation, sightseeing, camping, etc. required that there were beautiful mountains and waters, good air quality, suitable temperature and climate, sufficient water resources, etc.

As far as the ecosystem regulation services were concerned, water conservation involved rivers, creeks, streams, and ponds, which provided the sceneries of mountains and waters, as well as the sufficient water resources required by the industry of rural tourism; soil conservation mainly helped in maintaining the sceneries of mountains and waters through the alleviation of water loss and soil erosion; carbon sequestration of the ecosystem and oxygen supply improved air quality by absorption of carbon dioxide and increase in oxygen; climate adjustment ensured agreeable temperature and climate through the partial improvement of climate conditions; the anion supply in the forest ecosystem further improved air quality and allowed tourists to have better experiences. In consideration of the driving effects of ecosystem regulation services on rural tourism in terms of spatial layout, it could be discovered that it is difficult to explain the distribution features of rural tourist spots with a singular factor of service type, yet it would be easier to explain the same with dual factors. The development of rural tourism called for a great variety of actors. That is to say, the high-quality development of rural tourism could only be supported by the combined actions of different ecotourism regulation services.

**Figure 4:**
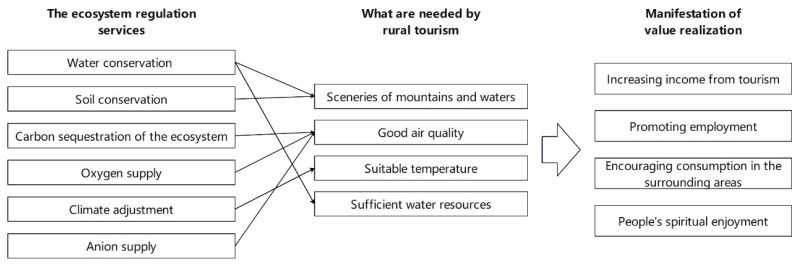
The supporting function of ecosystem regulation service to rural tourism.

For this reason, in view of the supply–demand relationship of industrial development (Figure 4), high-quality regulation services (one of the ecological products) provided by the ecosystem to human beings are closely relevant to the different factors needed in the development and operation of rural tourism and play a significant supportive role in the development of rural tourism. Meanwhile, the development of rural tourism will increase the income of people who work in the tourism industry in the relevant areas, create jobs, encourage consumption in the surrounding areas, effectively facilitate the implementation of the rural revitalization strategy, and, more importantly, enable the broad masses to get spiritual nourishment, enjoy the fruits of ecological civilization construction, and realize their social values, which further indicates that rural tourism is one of the important ways for the value realization of ecosystem services (ecological products) and for the support of ecosystem services for rural tourism development and green and sustainable development. For example, according to the statistics of the Culture and Tourism Development Committee of Wulong District, Wulong District, as one of the areas in the regions of study where rural tourism is densely distributed, received a total of 12,000,500 rural tourism tourists and realized consolidated income of RMB 2407 million in 2020, and Shizhu County clearly stated in its 14th Five-year Plan for Agricultural and Rural Modernization (2021–2025) that it will receive 5 million pastime agriculture tourism and rural tourism tourists in 2025 (it received 4.03 million in 2020), and it expects to raise the income from the relevant aspects to RMB 3 billion by that time.

## 4. Discussion

Rural tourism is one of the key measures during the implementation of the rural revitalization strategy [1,4,47], further indicating its feature of encouraging tourist consumption with superior natural ecological environments and conditions, and it is also an important way for the ecosystem to offer high-quality services to human beings and for the value realization of ecological products in money form. This paper, on the basis of existing studies, focuses on the special relationship between ecosystem regulation services and studies six types of ecosystem regulation services beyond the important factors, including the distribution of A-level scenic spots, locational conditions of transportation, and population density, that affect the spatial distribution of rural tourism, with the purpose of filling the gap in existing studies on the spatial relationship between rural tourism and ecosystem services and of offering new ideas of basic analysis for the formulation of better strategies for regional rural tourism development.

On the whole, in 2019, the spatial layout of rural tourist spots in the six districts and counties of the Wuling Mountains in southeastern Chongqing show a significant clustering trend, in line with the results of some existing studies [48]. Results of this paper also show that a singular factor of the ecosystem regulation services in the regions of study has weak driving effects on the spatial layout of rural tourism, which is different from what was expected. The main reason lies in that the distribution of rural tourism is more related to the overall environmental quality, and it is difficult for a single ecosystem regulation service to adequately support the development of rural tourism. However, the driving effects were obviously enhanced when two factors were combined, and the interaction of the combination of climate adjustment service and anion supply service exerted the most significant effect, which may be the reason for the 19.62% spatial distribution of rural tourist spots in the regions of study. In addition, the driving effects of the interactions between such combinations as climate adjustment and carbon sequestration of the ecological system, climate adjustment and oxygen supply, soil conservation and water conservation, and climate adjustment and water conservation are also strong on the spatial distribution of rural tourist spots within the regions of study. As further illustrated, it is difficult for a single ecosystem regulation service to exert an effective driving effect, but the combination of multiple ecosystem regulation services has a significant driving effect on the spatial distribution of rural tourism, which can be better explained as follows from the perspective of the supply–demand relationship during the development of the rural tourism industry: water conservation involved rivers, creeks, streams, and ponds, which provided the sceneries of mountains and waters, as well as sufficient water resources required by the industry of rural tourism; soil conservation mainly helped in maintaining the sceneries of mountains and waters through the alleviation of water loss and soil erosion; carbon sequestration of the ecosystem and oxygen supply improved air quality by absorption of carbon dioxide and increase in oxygen; climate adjustment ensured agreeable temperature and climate through the partial improvement of climate conditions; the anion supply in the forest ecosystem further improved air quality and allowed tourists to have better experiences. However, the rural tourism industry mainly attracts tourists to consume through the comprehensive conditions of pleasant climate, beautiful landscape, and high-quality air. The demand of the rural tourism industry for ecosystem regulation services is compound. In the process of site selection, the rural tourism industry tends to prefer the regions with multiple ecosystem regulation services.

Based on the results of this study, it is proposed in this paper that during the process of the special planning of rural tourism spots, comprehensive consideration should be given to the distribution of A-Level scenic spots, population density, and locational conditions of transportation [9,10,12,13]; the spatial layout of all types of ecosystem services in the region should be analyzed, priority should be given to the areas with more high-value services of climate adjustment and anion supply, and a rational layout should be planned in compliance with the policies for regional space-use control and economical and intensive land use under the preconditions that the original natural and ecological conditions must not be destroyed, so as to achieve the ultimate goals of fully tapping the natural and ecological strengths of the areas, further realizing the value of ecological products, and boosting rural revitalization.

Previous studies show a strong correlation between the spatial distribution of rural tourism and regional population, traffic, per capita income, and other social and economic factors, but because of the current limitations of data and technological means, such social and economic factors are not included in this study. The authors of this paper mainly probe into the driving effects of ecosystem services on the spatial layout of rural tourism and their supportive functions in the industrial development on the basis of an analysis of the six types of ecosystem regulation services, with the purpose of filling the gap in existing studies. Given that, how the interactions between ecosystem regulation services and social and economic factors drive the spatial distribution of rural tourism remains to be further analyzed in the future.

## 5. Conclusions

In this study, the authors set six districts and counties of the Wuling Mountains of southeastern Chongqing as the researched region. After ascertaining the spatial distribution of rural tourist spots within the researched region and six types of ecosystem regulation services, the authors selected geographic detector models to explore the driving effects of ecosystem regulation services on the spatial distribution of rural tourism and put forward relevant suggestions on the spatial planning of rural tourism in the next step, after taking into consideration of the results of analysis. The following conclusions are made: (1) the 1635 rural tourist spots within the researched region generally demonstrate significant features of aggregation, and they are densely scattered around scenic spots like the Xiannv Mountain National Forest Park of Wulong District and the Huangshui National Forest Park of Shizhu County; (2) among the six types of ecosystem regulation services, except for soil conservation services that have no obvious high-value areas of clustering, the other five types have obvious high-value areas, which are mainly distributed in forest ecosystems; (3) driven by a singular factor, the driving effects of the ecosystem regulation service factor on the spatial distribution of rural tourist spots are generally weak—in the event of dual factors, the driving effects are significantly enhanced, while the commonly shared driving effects of climate regulation and anion supply services are the most obvious; (4) from the perspective of the supply and demand relationship of industrial development, ecosystem services play an important role in supporting the development of rural tourism; (5) the next step in the spatial planning of rural tourism should be a comprehensive analysis of the impact of ecosystem regulation services, and guidance of the rational layout of industries on the premise of compliance with space-use control, as well as economical and intensive land use. In addition, conditions such as the economic basis and distribution of A-Level scenic spots need to be comprehensively taken into consideration.

## Figures and Tables

**Table 1:** Accounting method of ecosystem regulation services.

Index	Computing Method	Formula	Order Number
Water conservation	Equilibrium equation of water volume	$Q_{wr}=\overset{n}{\underset{i=1}{\sum }}A_{i}\times \left(P_{i}-R_{i}-ET_{i}\right)\times 10^{-3}\;$	(3)
Soil conservation	Revised universal soil loss equation (RUSLE)	$Q_{sr}=R\times K\times L\times S\times \left(1-C\times P\right)$	(4)
Ecosystem carbon sequestration	Carbon sequestration velocity method	$Q_{tCO_{2}}=M_{CO_{2}}/M_{C}\times \left(FCS+GSCS+WCS+CSCS\right)$	(5)
Oxygen supply	Coefficient transformation method	$Q_{op}=M_{O_{2}}/M_{CO_{2}}\times Q_{tCO_{2}}$	(6)
Climate regulation	Quantification through evapotranspiration	$A_{cm}=\left(\Sigma \Sigma ET\right)\times \rho_{w}\times L_{h}$	(7)
Anion supply	Calculation of the number of anions	$G_{\mathrm{Anion}}=1.314\times 10^{5}\times 10^{10}\times Q_{\mathrm{Anion}}\times A\times H/L$	(8)

**Table 2:** Interaction type determination rules.

Judgment Basis	Interaction Type
*q*(*X_i_* ∩ *X_j_*) < Min(*q*(*X_i_*), *q*(*X_j_*))	Nonlinear reduction
Min(*q*(*X_i_*), *q*(*X_j_*)) < *q*(*X_i_* ∩ *X_j_*) < Max(*q*(*X_i_*), *q*(*X_j_*))	Single-factor nonlinear reduction
*q*(*X_i_* ∩ *X_j_*) > Max(*q*(*X_i_*), *q*(*X_j_*))	Dual-factor enhancement
*q*(*X_i_* ∩ *X_j_*) > *q*(*X_i_*) + *q*(*X_j_*)	Nonlinear enhancement
*q*(*X_i_* ∩ *X_j_*) = *q*(*X_i_*) + *q*(*X_j_*)	Dual-driving factors independence

**Table 3:** Classification of driving factors of rural tourism spatial distribution.

	Driving Factor	Code	Factor Description
Driving factors of ecosystem regulation services	Water conservation	*X*_1_	The volume of water conservation in the unit area: to be calculated with Equation (3)
	Soil conservation	*X*_2_	The volume of water conservation in the unit area: to be calculated with Equation (4)
	Carbon sequestration of the ecosystem	*X*_3_	The volume of carbon sequestration of the ecosystem in the unit area: to be calculated with Equation (5)
	Oxygen supply	*X*_4_	The volume of oxygen supply in the unit area: to be calculated with Equation (6)
	Climate adjustment	*X*_5_	The volume of climate adjustment in the unit area: to be calculated with Equation (7)
	Anion supply	*X*_6_	The volume of anion supply in the unit area: to be calculated with Equation (8)

**Table 4:** Calculation results of nearest neighbor index of rural tourism.

${đ}_{0}\;(m)$	${đ}_{E}\;(m)$	NNI	Z Value	*p* Value
667.37	2363.45	0.28	−55.51	0.0000

Note: (1 − *p*) stands for the credibility of confidence interval.

**Table 5:** Differentiation and factor detection results.

	Driving Factor	Code	*q*	*p*
Driving factors of ecosystem regulation services	Water conservation	*X*_1_	0.0628	0
	Soil conservation	*X*_2_	0.0063	0.7493
	Carbon sequestration of the ecosystem	*X*_3_	0.0204	0.0255
	Oxygen supply	*X*_4_	0.0204	0.0255
	Climate adjustment	*X*_5_	0.0365	0
	Anion supply	*X*_6_	0.0333	0

**Table 6:** Interaction detection results.

	*X*_1_	*X*_2_	*X*_3_	*X*_4_	*X*_5_	*X*_6_
*X*_1_	0.0628					
*X*_2_	0.1712	0.0063				
*X*_3_	0.1150	0.0901	0.0204			
*X*_4_	0.1150	0.0901	0.0214	0.0204		
*X*_5_	0.1623	0.1257	0.1815	0.1815	0.0365	
*X*_6_	0.1190	0.0806	0.0902	0.0902	0.1962	0.0333

## Data Availability

Data available on request due to restrictions, e.g., privacy or ethical. The data presented in this study are available on request from the corresponding author. The data are not publicly available due to the confidentiality of data.
